# Kinetics of α-synuclein prions preceding neuropathological inclusions in multiple system atrophy

**DOI:** 10.1371/journal.ppat.1008222

**Published:** 2020-02-04

**Authors:** Amanda L. Woerman, Smita Patel, Sabeen A. Kazmi, Abby Oehler, Jisoo Lee, Daniel A. Mordes, Steven H. Olson, Stanley B. Prusiner

**Affiliations:** 1 Institute for Neurodegenerative Diseases, Weill Institute for Neurosciences, University of California, San Francisco, California, United States of America; 2 Department of Neurology, University of California, San Francisco, California, United States of America; 3 C.S. Kubik Laboratory for Neuropathology, Department of Pathology, Massachusetts General Hospital, Boston, Massachusetts, United States of America; 4 Department of Biochemistry and Biophysics, University of California, San Francisco, California, United States of America; Creighton University, UNITED STATES

## Abstract

Multiple system atrophy (MSA), a progressive neurodegenerative disease characterized by autonomic dysfunction and motor impairment, is caused by the self-templated misfolding of the protein α-synuclein. With no treatment currently available, we sought to characterize the spread of α-synuclein in a transgenic mouse model of MSA prion propagation to support drug discovery programs for synucleinopathies. Brain homogenates from MSA patient samples or mouse-passaged MSA were inoculated either by standard freehand injection or stereotactically into TgM83^+/-^ mice, which express human α-synuclein with the A53T mutation. Following disease onset, brains from the mice were tested for biologically active α-synuclein prions using a cell-based assay and examined for α-synuclein neuropathology. Inoculation studies using homogenates prepared from brain regions lacking detectable α-synuclein neuropathology transmitted neurological disease to mice. Terminal animals contained similar concentrations of α-synuclein prions; however, a time-course study where mice were terminated every five days through disease progression revealed that the kinetics of α-synuclein prion replication in the mice were variable. Stereotactic inoculation into the thalamus reduced variability in disease onset in the mice, although incubation times were consistent with standard inoculations. Using human samples with and without neuropathological lesions, we observed that α-synuclein prion formation precedes neuropathology in the brain, suggesting that disease in patients is not limited to brain regions containing neuropathological lesions.

## Author summary

The underlying cause of disease in a group of rapidly progressing neurodegenerative disorders called prion diseases is the misfolding of the prion protein (PrP) into a conformation that can self-template and spread disease throughout the brain. Diseases caused by this phenomenon include Creutzfeldt–Jakob disease (CJD), chronic wasting disease, and bovine spongiform encephalopathy (“mad cow” disease). In 2015, we demonstrated that this same mechanism is responsible for the neurodegenerative disease multiple system atrophy (MSA); however, the disease is caused by the misfolding of the protein α-synuclein rather than PrP. Having shown that α-synuclein prions in MSA patient samples exhibit a number of properties consistent with PrP prions in CJD patients, we sought to establish and define a rigorous transgenic mouse model of α-synuclein prion propagation to support ongoing drug discovery efforts for MSA therapeutics. In this study, we identified optimized methods for transmitting MSA in a transgenic mouse model of α-synuclein prion spreading and defined disease pathogenesis in these mice. These results are needed to properly evaluate compounds that may prevent α-synuclein prion spreading. We also showed that in both human and mouse brain, α-synuclein prion spreading precedes the formation of neuropathological lesions traditionally used to define disease, yielding new insights into the progression of MSA.

## Introduction

Protein misfolding diseases, or proteinopathies, are characterized by the misfolding of particular proteins, which often contain intrinsically disordered regions, into conformations with an increased β-sheet content. As a result, the protein develops the ability to serve as a self-template for additional protein misfolding [1]. Through this mechanism, a normal protein can become pathogenic, or capable of spreading disease in the central nervous system [2].

This mechanism was first proposed for the prion protein (PrP) [3]; in diseases including Creutzfeldt–Jakob disease (CJD), bovine spongiform encephalopathy, and scrapie, cellular PrP (PrP^C^) misfolds into a disease-causing isoform termed PrP^Sc^. Substantial work has subsequently shown that each of the diseases caused by PrP^Sc^ arises from the protein misfolding into a distinct conformation, or strain [4]. Combined, this groundbreaking work established the mechanism underlying a myriad of prion diseases and has more recently been shown to be true for several proteins: β-amyloid [5, 6], tau [7, 8], SOD-1 [9, 10], TDP-43 [11, 12], and α-synuclein [13, 14] misfold and self-propagate in cellular and animal models of disease.

Contributing to this growing field of investigation, we compared strain differences in the synucleinopathies Parkinson’s disease (PD) and multiple system atrophy (MSA), two movement disorders characterized by the neuropathological accumulation of α-synuclein in the brain. Using a cell-based assay, we demonstrated that distinct conformations of α-synuclein are present in PD and MSA patient samples [15]. Similar results have been reported by others [16, 17]. This important finding has significant clinical implications in light of drug discovery efforts to develop anti-PrP^Sc^ therapeutics: the compound IND24, which doubled the lifespan of mice inoculated with some strains of PrP^Sc^ [18], was ineffective in mice inoculated with CJD [19]. Given the evidence that prion strains are differentially susceptible to treatment with small molecules, we set out to characterize a transgenic (Tg) mouse model to be used by drug discovery programs specifically targeting the α-synuclein prion strain in MSA.

Earlier work showed that inoculating TgM83^+/-^ mice, which express the mutant human α-synuclein*A53T [20], with brain homogenate from deceased MSA patients induces neurological disease in ~120 days [13, 21]. Here, we demonstrate that homogenate from brain regions lacking glial cytoplasmic inclusions (GCIs), the hallmark neuropathology of MSA, are also capable of transmitting disease to TgM83^+/-^ mice. Similarly, in characterizing the kinetics of disease progression in inoculated animals, α-synuclein prions were also detected prior to the formation of neuropathological lesions. Our time-course experiment indicated that disease onset is variable across mice. To reduce variability, we compared stereotactic inoculations using mouse-passaged MSA brain homogenate with standard intrathalamic freehand injections. While initial disease onset remained consistent for both methods of inoculation, stereotactic inoculations into the thalamus substantially reduced experimental variability.

Together, these results not only provide key insights into the pathological mechanisms underlying MSA prion propagation in a valuable mouse model for drug discovery programs focused on α-synuclein prions, but also shed light on the clinical manifestations of disease pathogenesis in MSA patients.

## Results

### Alpha-synuclein prion spread precedes the formation of glial cytoplasmic inclusions in multiple system atrophy patients

Previously, we established a cellular assay using HEK293T cells to express human α-synuclein with the A53T mutation fused to yellow fluorescent protein (α-syn140*A53T–YFP) [14]. Culturing these cells in a 384-well plate with α-synuclein prions isolated from MSA patient samples induced α-synuclein aggregation, which was visualized by YFP-positive foci in the cytoplasm of the cells. Plates were imaged on an automated fluorescent microscope, and α-synuclein prion titer was measured by normalizing the total YFP fluorescence of all of the aggregates in an image to the cell count (× 10^3^ arbitrary units, A.U.). While characterizing this novel cell line, we unexpectedly found that we could detect α-synuclein prions from brain regions of MSA patients lacking the hallmark GCI pathology [14]. This finding led us to hypothesize that, in MSA patients, the formation and spreading of α-synuclein prions is an early step in the development of GCIs or the disease is not limited to the brain regions containing GCI pathology.

**Fig 1:**
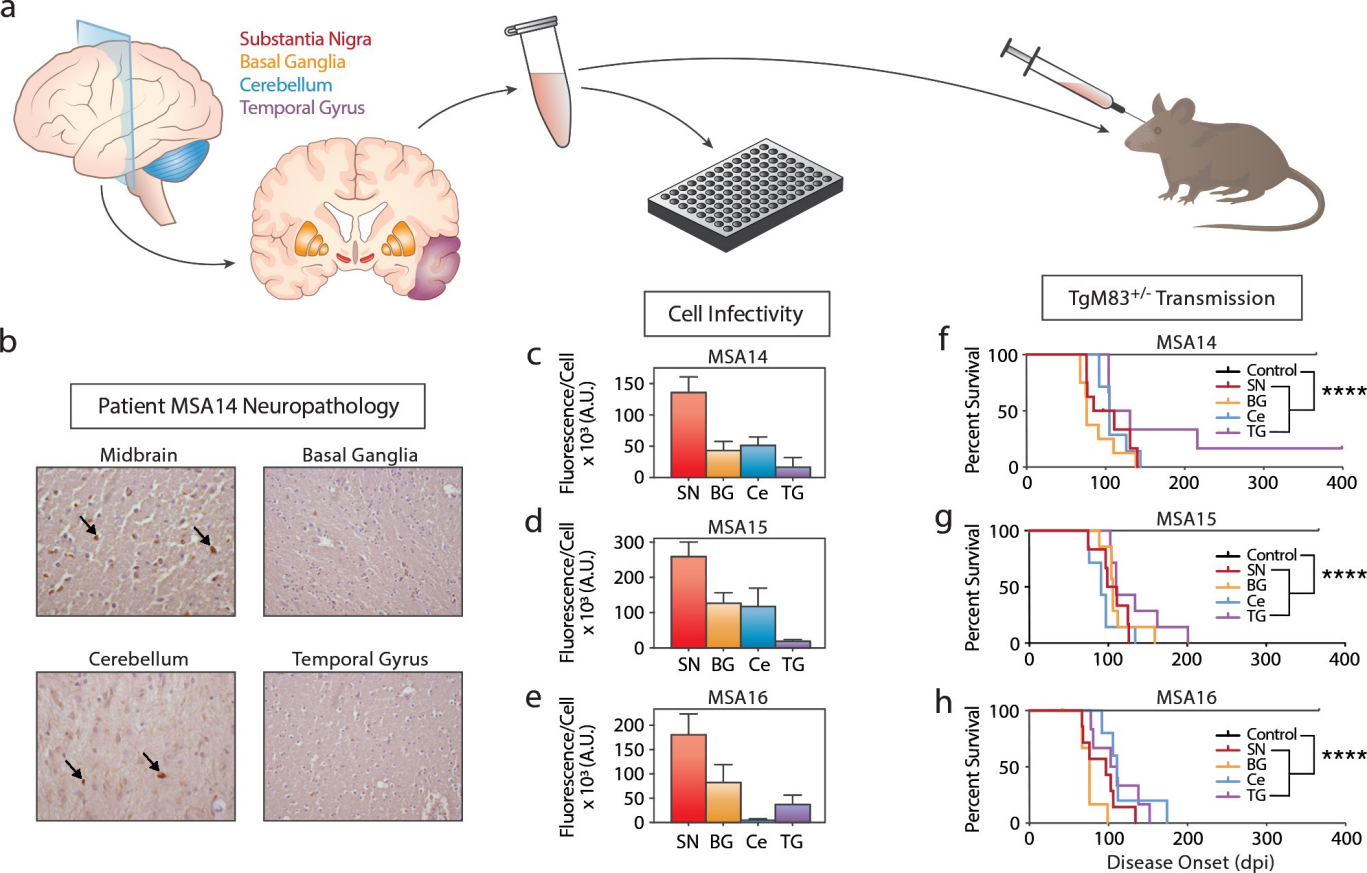
Brain regions lacking GCI pathology in MSA patients contain α-synuclein prions. (a) Frozen tissue from the substantia nigra (SN, red), basal ganglia (BG, orange), cerebellum (Ce, blue), or temporal gyrus (TG, purple) was collected from three MSA patients. The tissue was homogenized and tested for α-synuclein prions by cell assay, as well as mouse assay. (b) Representative images of glial cytoplasmic inclusion (GCI) pathology in the midbrain, basal ganglia, cerebellum, and temporal gyrus from patient MSA14. (c-e) Frozen tissue from the SN, BG, Ce, and TG from MSA patient samples MSA14, MSA15, and MSA16 were tested for the presence of α-synuclein prions in the α-syn140*A53T–YFP cell assay (× 10^3^ A.U.). Brain regions containing frequent GCI pathology (the SN) showed an elevated α-synuclein prion titer quantified by aggregate formation in the cells compared to brain regions lacking GCI pathologies (the TG). (f-h) Following isolation of α-synuclein prions by precipitation with phosphotungstic acid (PTA), the same patient samples were used to inoculate TgM83^+/-^ mice. Incubation times following MSA inoculation were compared with a PTA-precipitated control patient sample (shown in black). None of the control-inoculated mice developed neurological disease; however, mice inoculated with tissue from all MSA samples tested developed disease, even when tissue lacking GCI pathology was used. **** = *P* < 0.0001.

To test this hypothesis, we first sought to confirm our initial observation using a more sensitive clone of the original α-syn140*A53T–YFP cell line (Fig 1) [22]. (The initial α-syn140*A53T–YFP cell line could detect a minimum of 6 million molecules of α-synuclein [21], whereas the newer clone detects 1 million molecules [22].) Four brain regions (substantia nigra and surrounding midbrain, SN; basal ganglia, BG; cerebellum, Ce; and temporal gyrus, TG) from three MSA patients (MSA14, MSA15, and MSA16) were assessed for GCI density (S1 Table). In patient MSA14, GCIs were frequent in the SN and Ce, moderate in the BG, and rare in the TG (representative images in Fig 1B). Patient MSA15 had frequent GCIs in the Ce, moderate GCIs in the BG, but none in the SN or TG, and patient MSA16 had frequent GCIs in the SN and Ce, but none in the BG or TG. Brain homogenates prepared from the same brain regions were used to isolate α-synuclein prions via phosphotungstic acid (PTA) precipitation [23], and the resulting pellets were tested for infectivity in the α-syn140*A53T–YFP cells (Fig 1C–1E; S2 Table). Prior analysis has shown that aggregate formation in the α-syn140*A53T–YFP cells is reflective of α-synuclein prion titer [21]. Here, we observed differences in α-synuclein prion titer that were inconsistent with the scoring of GCI density in patients; however, all of the samples induced at least some aggregate formation in the cells.

In addition to the *in vitro* analysis, we tested all of the samples (12 total, 4 brain regions from 3 MSA patients) via *in vivo* transmission to the TgM83^+/-^ mouse model (Fig 1F–1H; S3 Table). Homozygous TgM83^+/+^ mice, which express human α-synuclein with the A53T mutation, develop spontaneous disease around one year of age [20], but hemizygous mice remain asymptomatic for over two years [13]. Alpha-synuclein prions were isolated by PTA-precipitation of homogenates from the 12 MSA samples and one control patient sample, and the purified and concentrated samples were inoculated intracranially into 10-week-old TgM83^+/-^ mice. Animals inoculated with the control sample remained asymptomatic for one year. However, mice inoculated with α-synuclein prions from all four brain regions from patients MSA14, MSA15, and MSA16 developed neurological disease (*P* < 0.0001). These results confirmed our hypothesis that disease-causing α-synuclein prions are widely distributed in the brains of MSA patients and may contribute to the clinical presentation of MSA.

### Symptomatic MSA-inoculated TgM83^+/-^ mice exhibit consistent α-synuclein prion concentrations

**Fig 2:**
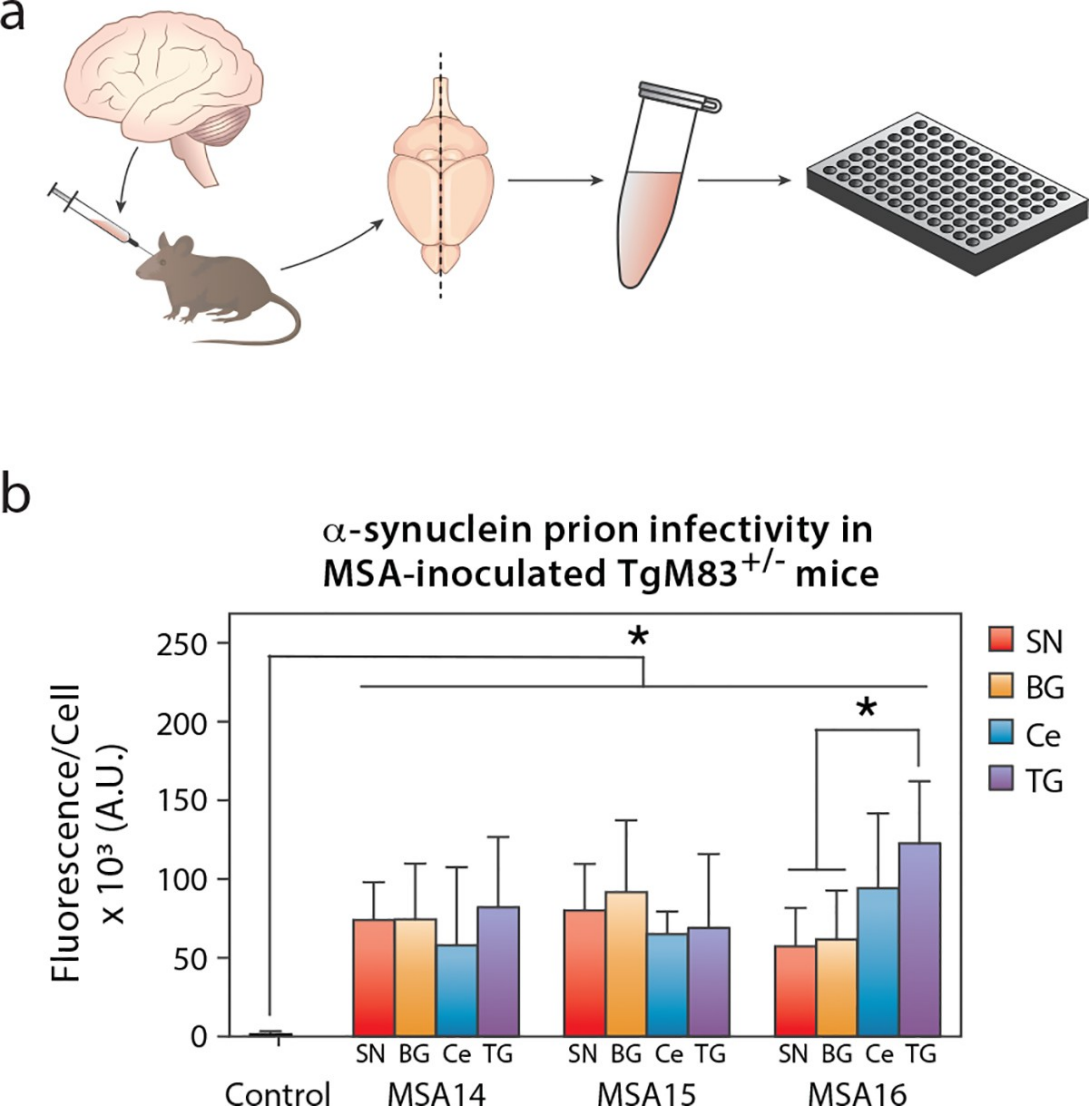
Terminal TgM83^+/-^ mice inoculated with MSA patient samples have similar concentrations of α-synuclein prions. TgM83^+/-^ mice were inoculated with phosphotungstic acid (PTA)-precipitated control or MSA patient samples. Mice were terminated after developing progressive neurological dysfunction, or 365 days post inoculation (dpi), and their brains were collected. (a) One half of the brain was flash frozen, homogenized, and tested for α-synuclein prions using the α-syn140*A53T–YFP cell assay (× 10^3^ A.U.). (b) Mice inoculated with the control patient sample were negative for α-synuclein prions (black), whereas mice inoculated with tissue from the substantia nigra and surrounding midbrain (SN), basal ganglia (BG), cerebellum (Ce), or temporal gyrus (TG) from three different MSA patients contained similar concentrations of α-synuclein prions at disease onset. * = *P* < 0.05.

**Fig 3:**
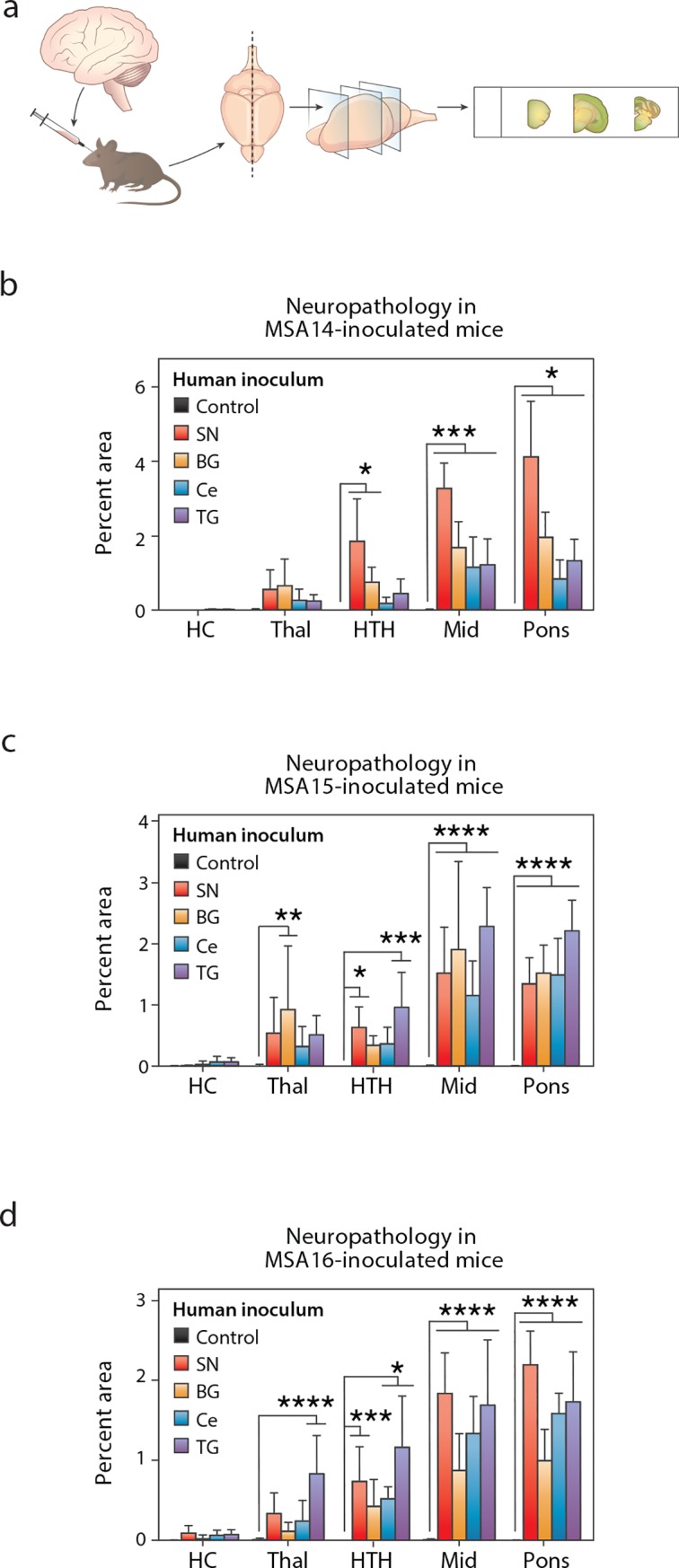
Terminal TgM83^+/-^ mice inoculated with MSA patient samples have similar neuropathological lesions. TgM83^+/-^ mice were inoculated with phosphotungstic acid (PTA)-precipitated control or MSA patient samples. Mice were terminated after developing progressive neurological dysfunction, or 365 days post inoculation (dpi), and their brains were collected. (a) One half of the brain was fixed in formalin, sectioned, and stained for phosphorylated α-synuclein pathology (EP1536Y primary antibody). (b-d) Neuropathology was measured in the hippocampus (HC), thalamus (Thal), hypothalamus (HTH), midbrain (Mid), and pons. Mice inoculated with the control patient sample (black) did not show immunolabeling. However, mice inoculated with any of the four brain regions from patients MSA14 (b), MSA15 (c), and MSA16 (d) developed neuropathological lesions in the Thal, HTH, Mid, and pons. * = *P* < 0.05. *** = *P* < 0.001. **** = *P* < 0.0001.

Following disease onset in the MSA-inoculated TgM83^+/-^ mice (Fig 1F–1H), animals were euthanized and their brains were collected for subsequent analysis (Figs 2 & 3). One half of each brain was flash frozen, homogenized, and tested for infectivity using the α-syn140*A53T–YFP cell assay (Fig 2; S4 Table). Brains from mice inoculated via standard freehand injection with the control patient sample did not infect the cells. In comparison, brains from mice inoculated with the brain region samples from patients MSA14 (*P* < 0.05), MSA15 (*P* < 0.01), and MSA16 (*P* < 0.01) showed a significant increase in cell infectivity. Comparing cell infectivity across the four brain regions by patient, no significant differences were detected in mice inoculated with homogenates from patients MSA14 or MSA15. However, mice inoculated with patient MSA16 TG tissue developed significantly more prions than mice inoculated with tissue from the SN (*P* < 0.01) and BG (*P* < 0.05).

In addition to testing infectivity, we assessed the other half-brain for phosphorylated α-synuclein immunostaining in the hippocampus (HC), thalamus (Thal), hypothalamus (HTH), midbrain (Mid), and pons (Fig 3; S1 Fig). Control-inoculated mice did not develop α-synuclein pathology. However, mice inoculated with homogenate from the three MSA patient samples developed pathology in the Thal, HTH, Mid, and pons, regardless of which brain region was used for the inoculum. Notably, the MSA-inoculated mice developed more extensive neuropathological lesions in the brainstem, with the Thal and HTH less affected. None of the mice developed α-synuclein inclusions in the HC. As previously reported, α-synuclein immunostaining in TgM83^+/-^ mice co-localized with labeling for p62 [22], a protein involved with degradation of aggregated α-synuclein in MSA [24]. These neuropathological changes were not observed in mice inoculated with the control patient sample.

### MSA prion propagation in TgM83^+/-^ mice is variable

**Fig 4:**
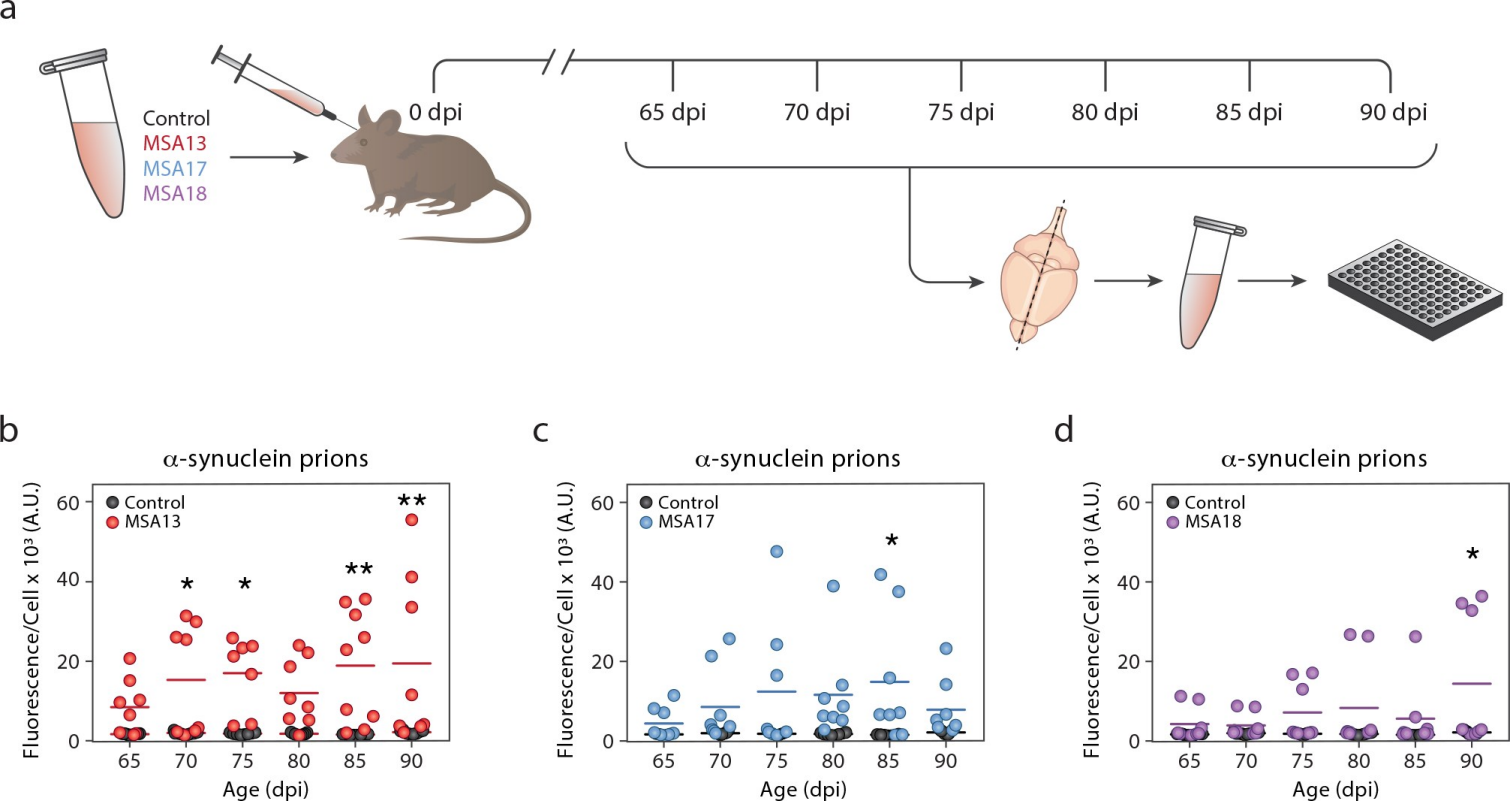
The rate of MSA prion propagation in TgM83^+/-^ mice is variable. TgM83^+/-^ mice were inoculated with brain homogenate from a control (black) or MSA patient sample (MSA13 in red, MSA17 in blue, and MSA18 in purple). (a) Eight mice from each inoculation group were terminated every 5 days, starting from 65 days post inoculation (dpi) to 90 dpi. Frozen half-brains were homogenized and tested for α-synuclein prions using the α-syn140*A53T–YFP cell assay (× 10^3^ A.U.). (b-d) None of the control-inoculated mice developed α-synuclein prions. The rate of α-synuclein prion formation varied across MSA-inoculated animals. * = *P* < 0.05; ** = *P* < 0.01.

Observing that the concentration of MSA prions in the brains of terminal TgM83^+/-^ mice is consistent, we wanted to measure the kinetics of α-synuclein prion propagation in these animals. To test this, we inoculated mice with brain homogenate from a control patient sample or one of three MSA patient samples (MSA13, MSA17, and MSA18) and collected 8 mice every 5 days from 65 to 90 dpi (Fig 4A). These samples were selected based on previous observations that all three induce neurological disease in TgM83^+/-^ mice following intracranial inoculation [25]. Given our finding that the average incubation time of MSA inoculation in TgM83^+/-^ mice is 120 days [21], we selected these time points to evaluate disease kinetics in pre-symptomatic animals. Frozen half-brains from all of the mice were individually homogenized and tested for infectivity in the α-syn140*A53T–YFP cell assay. All brains were tested in triplicate by two different individuals, and the average values for each mouse are plotted in Fig 4B–4D. None of the mice inoculated with the control patient sample contained α-synuclein prions. However, both the number of MSA-inoculated mice containing α-synuclein prions and the concentration of MSA prions in each animal were grossly inconsistent at each time point. Given the observed variability, most of the MSA-inoculated mice showed an insignificant difference from control animals across the six time points, with a handful of exceptions.

### MSA prion formation precedes α-synuclein neuropathology in TgM83^+/-^ mice

To understand the spatial distribution of disease spread in TgM83^+/-^ mice, we used formalin-fixed half-brains from mice collected at 90 dpi to assess phosphorylated α-synuclein neuropathology in the Thal, HTH, Mid, and pons (S2 Fig). Control-inoculated mice did not develop α-synuclein aggregates; however, brains from some of the MSA-inoculated animals did. Patient sample MSA13 induced the most pathology, with two mice showing robust staining in the Mid and pons but less in the Thal and HTH (S2B Fig). MSA17 induced the least pathology, with only one mouse showing a limited amount of immunolabeling in the Mid and pons (S2C Fig), and MSA18 produced intermediate results, with three mice developing marginal pathology in the Mid and pons (S2D Fig). Together, these findings suggest that in the TgM83^+/-^ mouse model, disease likely begins in the brainstem and spreads in a retrograde fashion into the Thal and HTH. This is also consistent with the increased density of α-synuclein neuropathology in the Mid and pons of terminal animals relative to the Thal and HTH (Fig 3).

**Fig 5:**
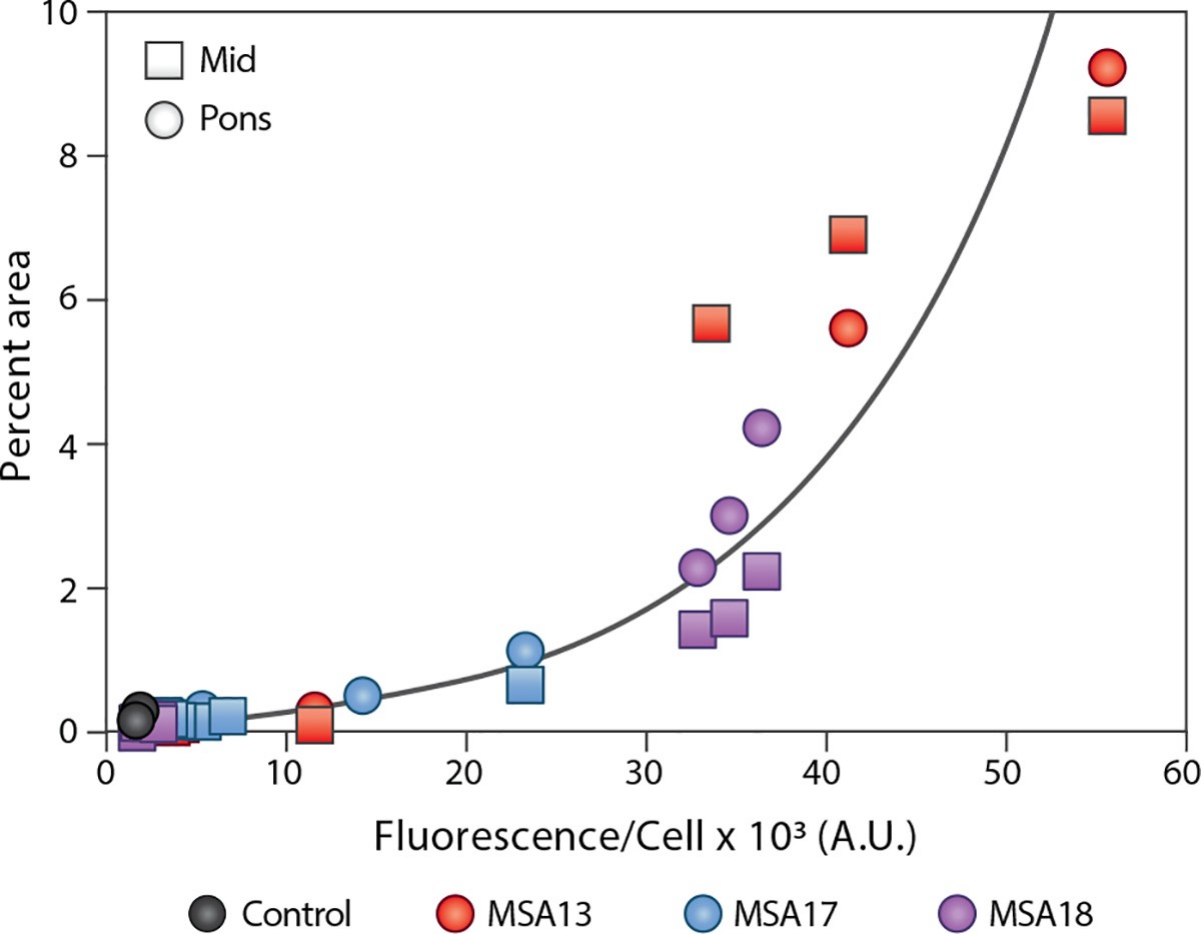
Alpha-synuclein prion propagation precedes neuropathology in TgM83^+/-^ mice. TgM83^+/-^ mice were inoculated with brain homogenate from a control (black) or an MSA patient sample (MSA13 in red, MSA17 in blue, and MSA18 in purple). Eight mice from each inoculation group were terminated 90 days post inoculation. The α-synuclein prion concentration, measured using frozen half-brains in the α-syn140*A53T–YFP cell assay (× 10^3^ A.U.), was compared with the phosphorylated α-synuclein neuropathology (EP1536Y primary antibody) measured from the midbrain (Mid; squares) and pons (circles) of each mouse. An exponential model (black line) was fit to the data, indicating α-synuclein prions are detectable in the brains of TgM83^+/-^ mice before aggregates can be seen by immunolabeling, and that propagation of α-synuclein prions is non-linear. R^2^ = 0.9395.

To compare the rate of α-synuclein prion formation with the rate of pathology development, we plotted the cell assay data from the mice collected at 90 dpi against the neuropathology measurements from the Mid and pons of the same animals (Fig 5). An exponential model was fit to the data (R^2^ = 0.9395), indicating that, similar to our findings using human tissue, α-synuclein prions are detected in the brain prior to the formation of pathology.

### Stereotactic inoculation does not alter disease *onset* in TgM83^+/-^ mice

**Fig 6:**
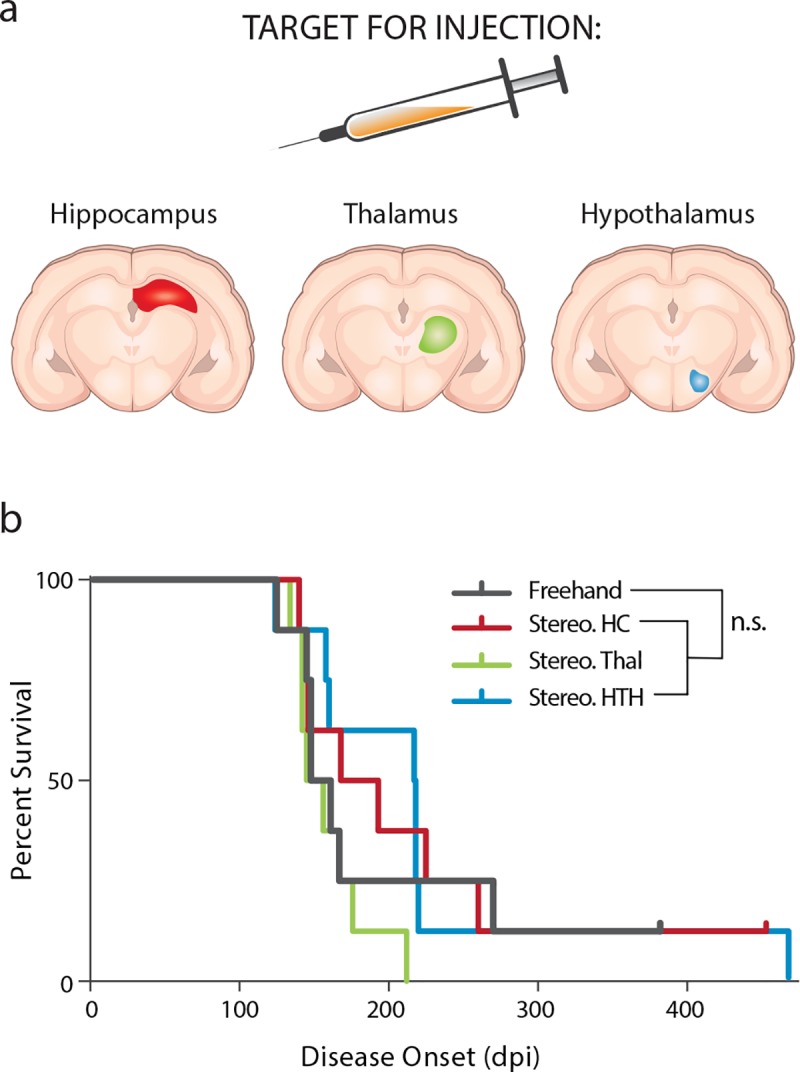
Stereotactic inoculation in TgM83^+/-^ mice does not alter disease onset. Secondary passage of an MSA patient sample (3 μL of a 5% homogenate) was used to inoculate TgM83^+/-^ mice either with a standard freehand injection (black) or by stereotactic inoculation into the hippocampus (Stereo. HC; red), thalamus (Stereo. Thal; green), or hypothalamus (Stereo. HTH; blue). (a) Schematic showing the experimental design. (b) Incubation times in TgM83^+/-^ mice were consistent across all four inoculation groups.

While freehand inoculation of PrP^Sc^ prions results in a fairly consistent onset of disease in mice, we observed substantial variability following freehand inoculation of MSA homogenates. Following this finding, we tested the hypothesis that a stereotactic inoculation, rather than the standard freehand injection used in PrP^Sc^ prion studies, would make disease onset more consistent (Fig 6). In this experiment, mice were inoculated with 3 μL of 5% mouse-passaged MSA homogenate. Stereotactic inoculations were done in the HC, Thal, or HTH (Fig 6A), and disease onset was compared to mice inoculated via standard intrathalamic injection (Fig 6B; S5 Table). No significant differences in incubation times were observed between mice inoculated using the standard protocol and mice inoculated stereotactically. Notably, despite the fact that mice in all four inoculation groups developed neurological disease around the same time, the substantial reduction in standard deviation observed following stereotactic inoculation into the thalamus suggests reduced variability in disease onset.

### Stereotactic inoculation does not alter disease *progression* in TgM83^+/-^ mice

**Fig 7:**
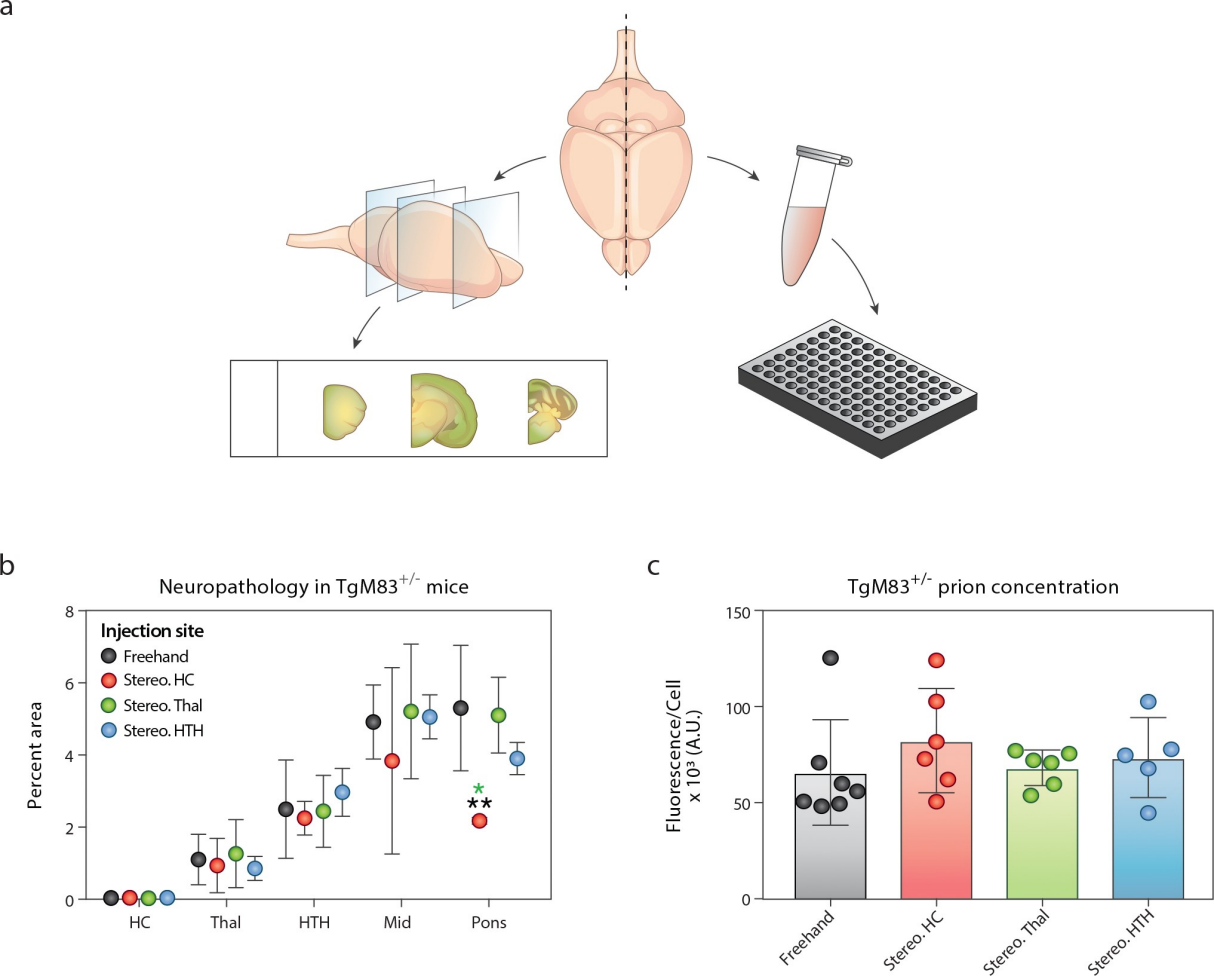
Alpha-synuclein prion concentration and neuropathology are unaltered by stereotactic inoculation in terminal TgM83^+/-^ mice. TgM83^+/-^ mice were inoculated with 3 μL of a 5% mouse-passaged MSA brain homogenate. Inoculations were performed either by standard freehand injection (black) or by stereotactic inoculation into the hippocampus (Stereo. HC; red), thalamus (Stereo. Thal; green), or hypothalamus (Stereo. HTH; blue). (a) After the mice developed progressive neurological disease, the animals were euthanized and their brains were harvested. One half of each brain was fixed in formalin, cut, and immunostained for phosphorylated α-synuclein neuropathology (data in panel b). The other half was flash frozen, homogenized, and tested for α-synuclein prions in the α-syn140*A53T–YFP cell assay (× 10^3^ A.U.; data in panel c). (b) Regardless of inoculation method or site, α-synuclein pathology measured in the HC, Thal, HTH, Mid, and pons was consistent across all four inoculation groups. (c) The α-synuclein prion concentration was consistent in terminal animals. * = *P* < 0.05; ** = *P* < 0.01.

To evaluate the effects of stereotactic inoculation on disease progression in TgM83^+/-^ mice further, we collected the brains from terminal animals and assessed half of the brain for α-synuclein neuropathology while the other half was tested in the α-syn140*A53T–YFP cell assay (Fig 7A). The MSA-induced α-synuclein neuropathology was independent of the stereotactic inoculation site (Fig 7B). Mice inoculated using the standard protocol developed pathology in the Thal, HTH, Mid, and pons, with the majority of pathology in the brainstem, but no α-synuclein accumulation was seen in the HC. Stereotactic inoculation into the HC, Thal, and HTH yielded a similar distribution. Unexpectedly, mice inoculated into the Thal showed minimal thalamic pathology, and mice inoculated into the HC had no detectable α-synuclein aggregates in this region. Additionally, the concentration of α-synuclein prions in the brains of terminal mice was consistent across all four inoculation groups (Fig 7C; S5 Table). Together, these results along with similar findings by others [26] present evidence of MSA strain tropism, a phenomenon previously demonstrated with PrP^Sc^ strains (reviewed in [4]). Some of this effect may be due to the distribution of transgene expression in TgM83^+/-^ mice or the cell type affected [16]; however, others have shown that a synthetic α-synuclein prion strain does induce HC pathology in these animals [27].

## Discussion

MSA has historically been recognized as a rapidly progressing movement disorder [28]. While the disease shares several features with PD, including α-synuclein pathology, MSA patients are poorly responsive to the levodopa [29] and deep brain stimulation (DBS) treatments [30] often used in PD patients. Unfortunately, there are currently no disease-modifying therapeutics available for MSA. To support ongoing and future drug discovery efforts, we report here a detailed characterization of disease progression in a mouse model that propagates α-synuclein prions from MSA patients. Importantly, we also demonstrate that in both humans and mice, α-synuclein prion formation precedes detectable neuropathology. This finding has critical implications for the clinical diagnosis and treatment of MSA, suggesting mechanisms for the progressive nature of neurodegeneration in patients and the onset of dementia later in disease.

Developing an effective therapeutic requires a well-understood animal model that successfully recapitulates the disease process. Using TgM83^+/-^ mice inoculated with brain homogenate from deceased MSA patients, we previously demonstrated that MSA induces profound neurological disease and death in these animals [13, 21]. Moreover, we have also shown that inoculations using α-synuclein aggregates isolated from MSA samples, devoid of nucleic acid, also transmit disease, indicating that MSA arises from the formation and spreading of α-synuclein prions [14]. Using cell-based assays to compare the biological and biochemical properties of α-synuclein prions in MSA versus pre-formed fibrils (PFFs) made from recombinant α-synuclein, we demonstrated that PFFs do not accurately model the α-synuclein pathogenesis seen in MSA [15].

Addressing the need to thoroughly evaluate disease progression in a mouse model propagating the MSA strain of α-synuclein, we performed a time-course experiment in TgM83^+/-^ mice. Measuring the α-synuclein prion concentration in the brains of mice collected between 65 and 90 dpi revealed that disease progression in the animals is variable. However, by comparing α-synuclein prion formation at 90 dpi with immunostaining of α-synuclein neuropathology, we observed that α-synuclein prion growth precedes the formation of detectable neuropathological lesions. It also suggests that α-synuclein prion spreading may exhibit exponential kinetics, an observation that is consistent with PrP^Sc^ spreading kinetics [31, 32].

Recognizing that important barriers to conducting drug discovery studies include the number of animals needed and the cost of long-term studies in mice, we sought to identify an inoculation paradigm to reduce variability in disease onset. Using mouse-passaged MSA, which we have shown contains an increased α-synuclein prion titer [14], we found that TgM83^+/-^ mice inoculated either by a standard freehand or stereotactic protocol developed disease at the same time. However, we also observed a substantial reduction in the variability of disease onset in mice receiving stereotactic inoculations into the Thal, suggesting this method may reduce the number of mice needed to properly power an efficacy study.

This experiment yielded an additional key observation: regardless of where the inoculum was delivered, TgM83^+/-^ mice always developed a similar degree of pathology in the Thal, HTH, Mid, and pons. One factor contributing to this finding is the spatial distribution of transgene expression. In this model, SNCA*A53T expression is regulated by the mouse *Prnp* promoter, meaning pathology is unlikely to develop in cells that do not express the protein substrate necessary for templating. However, recent work comparing two distinct strains of synthetic α-synuclein prions inoculated into TgM83^+/-^ mice found that fibrils developed in the absence of salt induced Lewy body-like pathology in several brain regions, including the HC, whereas PFFs formed in the presence of salt did not induce hippocampal pathology [27]. In light of these findings, the consistent neuropathological results in our stereotactic inoculation study demonstrate MSA prion strain tropism in TgM83^+/-^ mice.

A growing consensus recognizes that many MSA patients, reportedly up to 31%, develop mild to severe dementia throughout the course of disease [33–39]. Intriguingly, the data reported here demonstrate that α-synuclein prion formation precedes detectable neuropathology in both humans and mice. The observation that cortical tissue from terminal MSA patients, devoid of observable GCI pathology, transmits disease to TgM83^+/-^ mice indicates that disease is more widespread in the brains of MSA patients than previously recognized. However, diagnostic criteria for MSA is only reflective of the autonomic dysfunction and motor impairment that are present at disease onset [29].

The finding that α-synuclein prions precede GCIs also raises an important public health concern. Previously, we showed that stainless-steel wires incubated in brain homogenate from MSA patients and implanted into TgM83^+/-^ mice transmit disease [22]. This result raised concerns about the potential for iatrogenic transmission of MSA, particularly given the use of DBS to treat synucleinopathy patients. While a handful of studies have evaluated patient cohorts to investigate iatrogenic transmission, no evidence has been found [40–43]. In part, this may reflect the routes of transmission evaluated. Human-to-human transmission of CJD has notably occurred following injection of human growth hormone isolated from the pituitary glands of affected cadavers, transplants using either dura mater or corneas taken from CJD patients, and the use of contaminated neurosurgical instruments and electroencephalography electrodes [44]. However, it is unclear if these same tissue sources represent a risk for MSA transmission. Our finding that brain region samples from MSA patients lacking neuropathology still transmit disease argues that we cannot rely on GCI distribution to identify these risks. Instead, a thorough mapping of α-synuclein prion spread in the brains of MSA patients should be performed to determine which tissues present risks. This insight would enable epidemiological studies to evaluate the possibility of iatrogenic transmission of MSA.

Bringing MSA therapeutics into the clinic requires a better understanding of disease pathogenesis and spreading. The work reported here provides important insight into the disease kinetics of a mouse model of α-synuclein prion propagation for MSA, as well as the underlying pathobiology impacting a patient’s clinical presentation. Most notably, this work sheds light on the mechanism underlying a patient’s progression from a movement disorder to a movement disorder plus dementia. By recapitulating this process in a mouse model of disease, we have established an essential tool to support drug discovery efforts for MSA.

## Materials and methods

### Human tissue samples

Diagnosis of MSA was confirmed by bisecting fresh brains down the midline; one half was fixed in 10% neutral buffered formalin and coronally sectioned, and the other half was coronally sectioned before rapid freezing. Fixed sections were evaluated histologically using representative regions for a variety of neurodegenerative diseases. All sections were stained with Luxol fast blue and hematoxylin and eosin. Selected sections were also immunostained for α-synuclein, β-amyloid, and phosphorylated tau. An MSA diagnosis required identification of GCIs [29].

### Mice

All animals were maintained under standard environmental conditions with a 12:12-h light:dark cycle and free access to food and water. TgM83^+/-^ mice were generated by breeding TgM83^+/+^ mice [20] with B6C3F1 mice, all purchased from Jackson Laboratory.

### Inoculations

Frozen brain samples from humans or mice were homogenized in calcium- and magnesium-free 1× DPBS using the Omni Tissue Homogenizer (Omni International) to create a 10% (wt/vol) homogenate. Inoculations with phosphotungstic acid (PTA; Sigma) precipitated homogenates were performed using samples prepared as previously described [14, 45]. Inoculations using crude brain homogenates were performed by diluting samples to 1% using 5% (wt/vol) bovine serum albumin in 1× DPBS.

Ten-week-old TgM83^+/-^ mice were anesthetized with isoflurane prior to inoculation. Freehand inoculations with human patient samples were performed using 30 μL of either PTA-precipitated samples or 1% brain homogenate injected transcutaneously into the thalamus. Stereotactic inoculations were performed following a craniotomy by inoculating 3 μL of 5% (wt/vol) mouse-passaged MSA brain homogenate into the hippocampus (HC; Bregma: -2.3 mm, lateral: +1.8 mm, depth: -1.88 mm), thalamus (Thal; Bregma: -2.3 mm, lateral: +1.65 mm, depth: -3.6 mm), or hypothalamus (HTH; Bregma: -2.3 mm, lateral: +1 mm, depth: -5.15 mm). All animals undergoing stereotaxic inoculation were administered bupivacaine <8 mg/kg at the injection site, buprenorphine 0.1 mg/kg subcutaneously, and meloxicam 7.5 mg/kg subcutaneously. Stereotaxic inoculations were compared to freehand inoculations also performed with 3 μL of 5% mouse-passaged MSA homogenate.

Following inoculation, all mice were examined at least daily for any abnormal clinical sign(s) using a systematic neurological protocol that included assessment of ambulation, lack or presence of hind-leg clasping, presence or absence of ataxia, and testing for the righting reflex. Animals displaying two or more clinical signs were identified, and if their clinical condition did not change or deteriorated within 24 hours, then the animals were euthanized. Control-inoculated and asymptomatic mice were euthanized >365 days post inoculation (dpi). For the time-course experiment, 8 mice (4 male and 4 female) were collected every 5 days from 65–90 dpi. Following euthanasia, the brain was removed and bisected down the midline. The left hemisphere was frozen for biochemical analysis, and the right hemisphere was fixed in formalin for neuropathological assessment.

### Alpha-synuclein prion quantification assay

Aggregated protein was isolated from 10% (wt/vol) patient samples or mouse brain homogenates using PTA. Isolated protein pellets were diluted 1:10 in 1× DPBS before testing in the α-synuclein prion quantification assay previously described [14]. Briefly, human embryonic kidney (HEK293T) cells expressing α-syn140*A53T–YFP were plated at a density of 2,500 cells/well (brain region and time-course studies) or 3,000 cells/well (stereotactic inoculation samples). Images of cells incubated with human or mouse samples were collected using the IN Cell Analyzer 6000 (GE Healthcare). DAPI and FITC channels were used to collect two images from five different regions in each well. Each set of images was analyzed using the IN Cell Developer software with an algorithm created to detect intracellular aggregates in living cells, quantified as total fluorescence per cell (× 10^3^, arbitrary units, A.U.).

### Immunohistochemistry and neuropathology

Mouse brains were fixed in 10% (vol/vol) formalin, processed, embedded, and sectioned as previously described [25]. Brains were cut into four sections prior to processing through graded alcohols, clearing with xylene, infiltrating with paraffin, and embedding. After deparaffinization, sections were exposed to heat-mediated antigen retrieval with citrate buffer (0.1 M, pH 6) for 20 min. Slides were stained overnight at room temperature after blocking in 10% (vol/vol) normal goat serum using EP1536Y (pS129 α-synuclein; 1:1,000; Abcam), p62 (Anti-SQSTM1; 1:1,000; Abcam), and glial fibrillary acidic protein (GFAP; 1:500; Abcam) primary antibodies. Secondary antibodies conjugated to AlexaFluor 488, 568, or 647 (Thermo Fisher) were used to detect immunolabeling.

Slides were imaged using the Zeiss AxioScan.Z1. Digital images were analyzed using the Zen Analysis software package (Zeiss). To quantify α-synuclein neuropathology, a pixel intensity threshold was determined using a positive control slide and was then applied to all slides. Regions of interest were drawn around the HC, Thal, HTH, midbrain (Mid), and pons. The percentage of pixels positive for staining in each region was determined.

### Statistical analysis

Data are presented as mean ± standard deviation. Statistical analysis of the Kaplan-Meier curves in the TgM83^+/-^ mice was done using a log-rank Mantel-Cox test. Cell assay data collected from the MSA patient sample brain regions, as well as from the mice receiving stereotactic inoculations, were analyzed using a one-way ANOVA with a Tukey multiple comparison post hoc test. Data from the time-course experiment, as well as neuropathology data from the brain region inoculations and the stereotactic inoculation study, were analyzed using a two-way ANOVA with a Dunnett multiple comparison post hoc test. An exponential model was fit to the data comparing cell infectivity with neuropathology in the 90-day time point from the time-course experiment. Significance was determined with a *P* value < 0.05.

### Ethics statement

Animals were maintained in an AAALAC-accredited facility in compliance with the *Guide for the Care and Use of Laboratory Animals*. All procedures used in this study were approved by the University of California, San Francisco, Institutional Animal Care and Use Committee (IACUC) under approved protocol AN16110-03D, “Incubation Periods and Behavioral Characterization of Animals Infected with Prions.” Frozen brain tissue samples from neuropathologically confirmed cases of MSA were provided by the Massachusetts Alzheimer’s Disease Research Center. Control patient tissue was provided by Dr. Martin Ingelsson (Uppsala University). UCSF Institutional Review Board approval was not required for this study. All human samples were anonymized.

## Supporting information

S1 Fig Phosphorylated α-synuclein pathology co-localizes with p62 in MSA-inoculated TgM83^+/-^ mice. TgM83^+/-^ mice were inoculated with phosphotungstic acid (PTA)-precipitated control or MSA patient samples. Mice were terminated after developing progressive neurological dysfunction, or 365 days post inoculation (dpi), and their brains were collected. One half of the brain was fixed in formalin, sectioned, and stained for phosphorylated α-synuclein pathology (EP1536Y primary antibody), p62, and astrogliosis (glial fibrillary acidic protein, GFAP). Representative micrographs show co-localization of phosphorylated α-synuclein (green) with p62 (violet; merge shown in white) surrounded by astrogliosis (red) in the brainstem of a mouse inoculated with MSA16 TG tissue (right). These lesions are absent in the control-inoculated animals (left). DAPI in blue. Scale bar = 50 μm.
(TIF)

S2 Fig Alpha-synuclein pathology is inconsistent in TgM83^+/-^ mice 90 days after MSA inoculation. TgM83^+/-^ mice were inoculated with brain homogenate from a control (in black) or an MSA patient sample (MSA13 in red, MSA17 in blue, and MSA18 in purple). Eight mice from each inoculation group were terminated 90 days post inoculation. Fixed half-brains were cut and stained for phosphorylated α-synuclein (EP1536Y primary antibody), and the percent area containing pathology was measured in the thalamus (Thal), hypothalamus (HTH), midbrain (Mid), and pons. (a) Graphic representation of the experiment. (b-d) Neuropathology measured in mice inoculated with control sample, (b) MSA13, (c) MSA17, or (d) MSA18. None of the control-inoculated mice developed α-synuclein pathology; however, both the presence and amount of α-synuclein accumulation in the MSA-inoculated mice were inconsistent. * = *P* < 0.05; ** = *P* < 0.01.
(TIF)

S1 Table Semiquantitation of GCI density in MSA patient samp. (PDF)

S2 Table SA prion propagation in α-syn140*A53T–YFP cells. (PDF)

S3 Table MSA transmission to TgM83^+/-^mice. (PDF)

S4 Table MSA prion concentration in symptomatic TgM83^+/-^mice. (PDF)

S5 Table MSA transmission to TgM83^+/-^mice. (PDF)
