# Supplementary figures and images for: Kinetics of α-synuclein prions preceding neuropathological inclusions in multiple system atrophy
Source: PLoS Pathog. 2020 Feb 4;16(2):e1008222. doi: 10.1371/journal.ppat.1008222 (PMC6999861; doi:10.1371/journal.ppat.1008222)

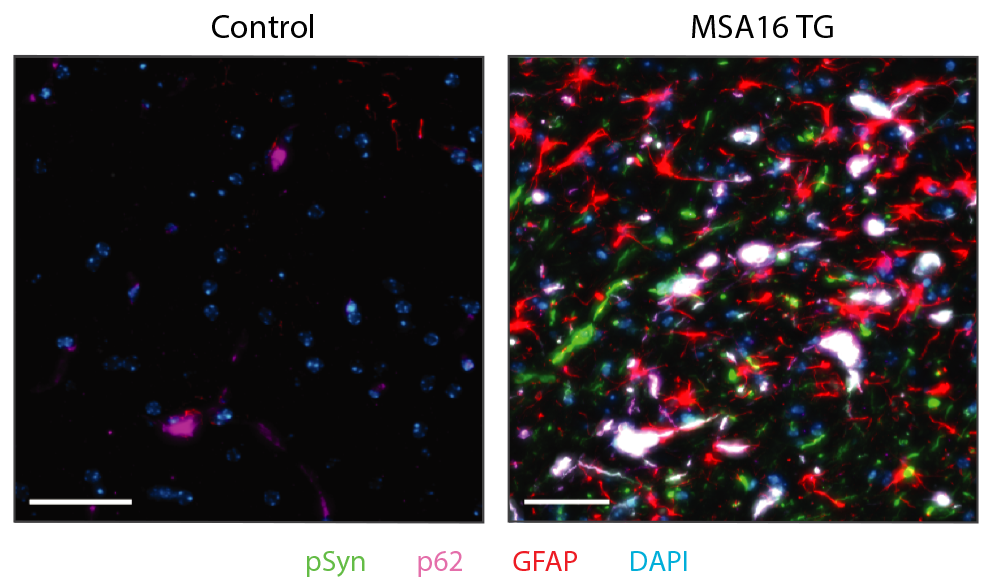

Supplement: S1 Fig — TgM83+/- mice were inoculated with phosphotungstic acid (PTA)-precipitated control or MSA patient samples. Mice were terminated after developing progressive neurological dysfunction, or 365 days post inoculation (dpi), and their brains were collected. One half of the brain was fixed in formalin, sectioned, and stained for phosphorylated α-synuclein pathology (EP1536Y primary antibody), p62, and astrogliosis (glial fibrillary acidic protein, GFAP). Representative micrographs show co-localization of phosphorylated α-synuclein (green) with p62 (violet; merge shown in white) surrounded by astrogliosis (red) in the brainstem of a mouse inoculated with MSA16 TG tissue (right). These lesions are absent in the control-inoculated animals (left). DAPI in blue. Scale bar = 50 μm. (TIF) [file ppat.1008222.s001.tif]

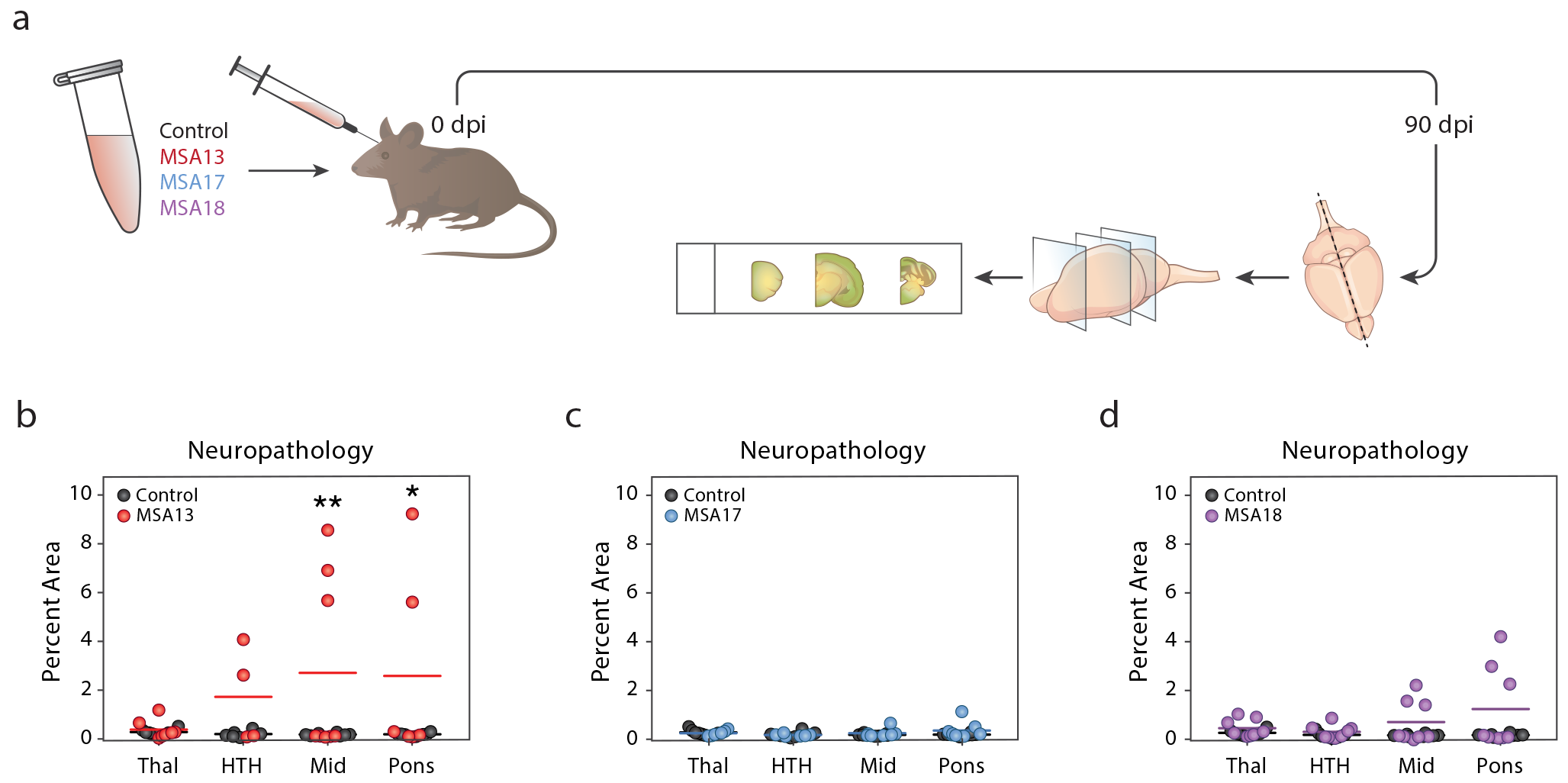

Supplement: S2 Fig — TgM83+/- mice were inoculated with brain homogenate from a control (in black) or an MSA patient sample (MSA13 in red, MSA17 in blue, and MSA18 in purple). Eight mice from each inoculation group were terminated 90 days post inoculation. Fixed half-brains were cut and stained for phosphorylated α-synuclein (EP1536Y primary antibody), and the percent area containing pathology was measured in the thalamus (Thal), hypothalamus (HTH), midbrain (Mid), and pons. (a) Graphic representation of the experiment. (b-d) Neuropathology measured in mice inoculated with control sample, (b) MSA13, (c) MSA17, or (d) MSA18. None of the control-inoculated mice developed α-synuclein pathology; however, both the presence and amount of α-synuclein accumulation in the MSA-inoculated mice were inconsistent. * = P < 0.05; ** = P < 0.01. (TIF) [file ppat.1008222.s002.tif]
