# Supplementary material for: Kinetics of α-synuclein prions preceding neuropathological inclusions in multiple system atrophy
Source: PLoS Pathog. 2020 Feb 4;16(2):e1008222. doi: 10.1371/journal.ppat.1008222 (PMC6999861; doi:10.1371/journal.ppat.1008222)
Supplement: S1 Table — (PDF) [file ppat.1008222.s003.pdf]

Table S1. Semiquantitation of GCI density in MSA patient samples.

Patient sample	Brain region			
	**Substantia nigra**	**Basal ganglia**	**Cerebellum**	**Temporal gyrus**
MSA14	Frequent	Moderate	Frequent	Rare
MSA15	None	Moderate	Frequent	None
MSA16	Frequent	None	Frequent	None
