# Supplementary material for: Kinetics of α-synuclein prions preceding neuropathological inclusions in multiple system atrophy
Source: PLoS Pathog. 2020 Feb 4;16(2):e1008222. doi: 10.1371/journal.ppat.1008222 (PMC6999861; doi:10.1371/journal.ppat.1008222)
Supplement: S2 Table — (PDF) [file ppat.1008222.s004.pdf]

Table S2. MSA prion propagation in α-syn140*A53T–YFP cells.

Patient sample	Cell infectivity (× 10^3 A.U.)*			
	Substantia nigra	Basal ganglia	Cerebellum	Temporal gyrus
MSA14	140 ± 24	44 ± 14	52 ± 13	17 ± 15
MSA15	260 ± 40	130 ± 28	120 ± 51	20 ± 3.7
MSA16	181 ± 42	83 ± 36	6.0 ± 1.8	38 ± 18

**Measurements made from five images per well, n = 6 wells. Phosphotungstic acid (PTA)-precipitated samples were diluted in DPBS 1:10 before testing on α-syn140*A53T–YFP cells.*
