# Supplementary material for: Kinetics of α-synuclein prions preceding neuropathological inclusions in multiple system atrophy
Source: PLoS Pathog. 2020 Feb 4;16(2):e1008222. doi: 10.1371/journal.ppat.1008222 (PMC6999861; doi:10.1371/journal.ppat.1008222)
Supplement: S3 Table — (PDF) [file ppat.1008222.s005.pdf]

Table S3. MSA transmission to TgM83$^{+/-}$ mice.

Patient sample	Incubation time (days post inoculation)			
	**Substantia nigra**	**Basal ganglia**	**Cerebellum**	**Temporal gyrus**
MSA14	99 ± 28	88 ± 25	110 ± 19	132 ± 48
MSA15	106 ± 19	111 ± 22	94 ± 20	132 ± 37
MSA16	93 ± 24	77 ± 12	119 ± 32	109 ± 27
