# Supplementary material for: Kinetics of α-synuclein prions preceding neuropathological inclusions in multiple system atrophy
Source: PLoS Pathog. 2020 Feb 4;16(2):e1008222. doi: 10.1371/journal.ppat.1008222 (PMC6999861; doi:10.1371/journal.ppat.1008222)
Supplement: S4 Table — (PDF) [file ppat.1008222.s006.pdf]

Table S4. MSA prion concentration in symptomatic TgM83$^{+/-}$ mice.

Patient sample	Cell infectivity (× 10^3 A.U.)*			
	Substantia nigra	Basal ganglia	Cerebellum	Temporal gyrus
MSA14	75 ± 23	76 ± 34	59 ± 48	83 ± 43
MSA15	81 ± 28	93 ± 44	66 ± 13	70 ± 46
MSA16	59 ± 23	63 ± 30	95 ± 46	124 ± 38

**Measurements made from five images per well, n = 6 wells. Phosphotungstic acid (PTA)-precipitated samples were diluted in DPBS 1:10 before testing on α-syn140*A53T–YFP cells.*
