# Supplementary material for: Kinetics of α-synuclein prions preceding neuropathological inclusions in multiple system atrophy
Source: PLoS Pathog. 2020 Feb 4;16(2):e1008222. doi: 10.1371/journal.ppat.1008222 (PMC6999861; doi:10.1371/journal.ppat.1008222)
Supplement: S5 Table — (PDF) [file ppat.1008222.s007.pdf]

Table S5. MSA transmission to TgM83$^{+/-}$ mice.

Inoculation site	Incubation time (days post inoculation)	Cell infectivity (× 10^3 A.U.)*
Standard freehand	166 ± 48	66 ± 27
Hippocampus	182 ± 46	82 ± 27
Thalamus	159 ± 26	68 ± 9.3
Hypothalamus	223 ± 106	73 ± 21

**Measurements made from five images per well, n = 6 wells. Phosphotungstic acid (PTA)-precipitated samples were diluted in DPBS 1:10 before testing on α-syn140*A53T–YFP cells.*
